# Molecular magnetic resonance imaging (MRI) of inflamed myocardium using ferucarbotran in patients with acute myocardial infarction

**DOI:** 10.1186/1532-429X-13-S1-P149

**Published:** 2011-02-02

**Authors:** Ali Yilmaz, Sabine Rösch, Karin Klingel, Reinhard Kandolf, Xavier Helluy, Karl-Heinz Hiller, Peter M Jakob, Udo Sechtem

**Affiliations:** 1 Robert-Bosch-Krankenhaus, Stuttgart, Germany; 2 Department of Molecular Pathology, Tübingen, Germany; 3 University of Würzburg, Würzburg, Germany

## Introduction

Superparamagnetic iron oxide nanoparticle (SPIO)-based molecular imaging agents targeting macrophages have been developed and successfully applied in animal models of myocardial infarction.

## Purpose

The purpose of this clinical trial was to investigate whether molecular magnetic resonance imaging (MRI) of macrophages using ferucarbotran (Resovist®) allows improved visualization of the myocardial (peri-)infarct zone compared to conventional gadolinium-based necrosis/fibrosis imaging in patients with acute myocardial infarction.

## Methods

The clinical study NIMINI-1 was performed as a prospective, non-randomised, non-blinded, single agent phase III clinical trial. Twenty patients who had experienced either an acute ST-elevation or non-ST-elevation myocardial infarction (STEMI/NSTEMI) were included to this study. Following coronary angiography, a first baseline cardiovascular magnetic resonance (CMR) study (pre-SPIO) was performed within seven days after onset of cardiac symptoms. A second CMR study (post-SPIO) was performed either 10min, 4h, 24h or 48h after ferucarbotran administration. The CMR studies comprised cine-CMR, T2-weighted “edema” imaging, T2*-weighted cardiac imaging and T1-weighted late-gadolinium-enhancement (LGE) imaging.

## Results

The median extent of short-axis in-plane LGE was 28% (IQR 19-31%). Following Resovist® administration the median extent of short-axis in-plane T2*-weighted hypoenhancement (suggestive of intramyocardial hemorrhage and/or SPIO accumulation) was 0% (IQR 0-9%; p=0.68 compared to pre-SPIO). A significant in-slice increase (>3%) in the extent of T2*-weighted “hypoenhancement” (post-SPIO compared to pre-SPIO) was seen in 6/16 patients (38%). However, no patient demonstrated “hypoenhancement” in T2*-weighted images following Resovist® administration that exceeded the area of LGE.

## Conclusions

T2/T2*-weighted MRI aiming at non-invasive myocardial macrophage imaging using the approved dose of ferucarbotran does not allow improved visualization of the myocardial (peri-) infarct zone compared to conventional gadolinium-based necrosis/fibrosis imaging.

